# Case report: Salivary duct carcinoma in a patient with a germline *CDH1* pathogenic variant - expanding the spectrum of hereditary cancer predisposition syndromes

**DOI:** 10.3389/fonc.2024.1372382

**Published:** 2024-04-08

**Authors:** Nidhi Desai, Emilian Racila, Naomi Fujioka, Arjun Gupta, Emmanuel S. Antonarakis

**Affiliations:** ^1^ Division of Hematology, Oncology & Transplantation, University of Minnesota, Minneapolis, MN, United States; ^2^ Department of Laboratory Medicine and Pathology, University of Minnesota, Minneapolis, MN, United States

**Keywords:** *CDH1*, germline, pathogenic variant, salivary duct carcinoma, cancer

## Abstract

**Introduction:**

Recently, an entity known as salivary duct carcinoma with rhabdoid features (SDC-RF) has been associated with somatic *CDH1* mutations. Here we present the first known case report of conventional SDC occurring in the setting of a germline *CDH1* pathogenic variant accompanied by a somatic loss of heterozygosity at the *CDH1* locus.

**Case discussion:**

A 67-year-old man presented with chest and back pain and was found to have osteolytic lesions in the sternum and lumbar spine. Vertebral bone biopsies were positive for metastatic carcinoma of unknown primary. A molecular profiling assay consisting of both whole-exome next-generation sequencing (NGS) as well as immunohistochemistry (IHC) for select clinically-relevant proteins performed on the bone biopsy suggested a triple-negative (ER/PR/ERBB2 negative, by IHC), androgen receptor (AR IHC) positive tumor profile. Additionally, the assay uncovered a coding mutation in the *CDH1* gene (c.1792C>T, p.R598*) with genomic loss of the second *CDH1* allele. Germline testing returned positive for a heterozygous *CDH1* pathogenic variant. PET-CT revealed a tumor in the neck suggestive of the primary malignancy consistent with that of salivary gland origin. The patient was initially treated with carboplatin and paclitaxel, then pembrolizumab, and finally with AR-directed therapy using leuprolide and enzalutamide. These treatments were not successful, and the patient eventually succumbed to his disease.

**Conclusion:**

Molecular testing revealed that our patient had bi-allelic inactivation of the *CDH1* gene. We believe our patient developed a somatic mutation in addition to his preexisting germline *CDH1* mutation that ultimately predisposed him to SDC. While previous studies have found somatic *CDH1* pathogenic variants in SDC-RF, our patient was found to have a germline *CDH1* pathogenic variant in the setting of conventional SDC, without rhabdoid features. This case provokes questions regarding tumor genetics and molecular profiling of SDC in patients with germline *CDH1* pathogenic variants. Moreover, this case supports the notion that SDC may be the salivary counterpart of other malignancies associated with germline *CDH1* pathogenic variants and may possibly expand the spectrum of tumors that arise in this familial cancer-predisposition syndrome.

## Introduction

E-cadherin, the protein product of the *CDH1* gene, is a trans-membrane glycoprotein involved in calcium-dependent cell-to-cell adhesion and plays a key role in epithelial cell behavior (1, 2). Deregulation of E-cadherin secondary to loss of the *CDH1* gene allows for cancer invasion and metastasis due to the loss of cell adhesion and acquisition of cell motility (3). Germline pathogenic variants in *CDH1* are mostly associated with diffuse gastric cancer (DGC) and lobular breast carcinoma (LBC). In *CDH1* carriers, the overall lifetime risk of DGC is 42% in men and 33% in women, and the cumulative incidence of LBC is around 42% in women (3, 4).

More recently, salivary duct carcinoma (SDC) has been associated with somatic *CDH1* mutations. SDC represents less than 2% of all salivary gland cancers, which have been found to have incidence rates of 0.1 to 2.7 per 100,000 individuals worldwide (5). Given that SDC is an extremely rare malignancy, limited literature exists on its association with *CDH1*. Rooper et al. found that 86% of salivary duct carcinomas with rhabdoid features (SDC-RF) had somatic *CDH1* alterations with corresponding abnormal expression or complete loss of E-cadherin protein in 78% of tumors (6). Likewise, Kusafuka et al. found the presence of *CDH1* alterations in 72% of all SDC-RF (7). Here, we present the clinical and genomic features of a patient with SDC without rhabdoid features in the setting of a germline *CDH1* pathogenic variant.

## Case description

A 67-year-old white male with a past medical history of hypertension, hyperlipidemia, and coronary artery disease (CAD) presented with chest and back pain. He had no significant history of alcohol or tobacco use disorder. Due to his history of CAD, there was initially concern for unstable angina as the underlying cause of his chest pain. However further workup revealed osteolytic lesions in the sternum, ribs, lumbar, and thoracic spine as the etiology of his pain. There was no evidence of a visceral primary tumor on computed tomography (CT) of the chest, abdomen, and pelvis. Physical examination was largely unremarkable other than the presence of nontender, left-sided, posterior cervical lymphadenopathy. He had no hepatosplenomegaly.

**Figure 1:**
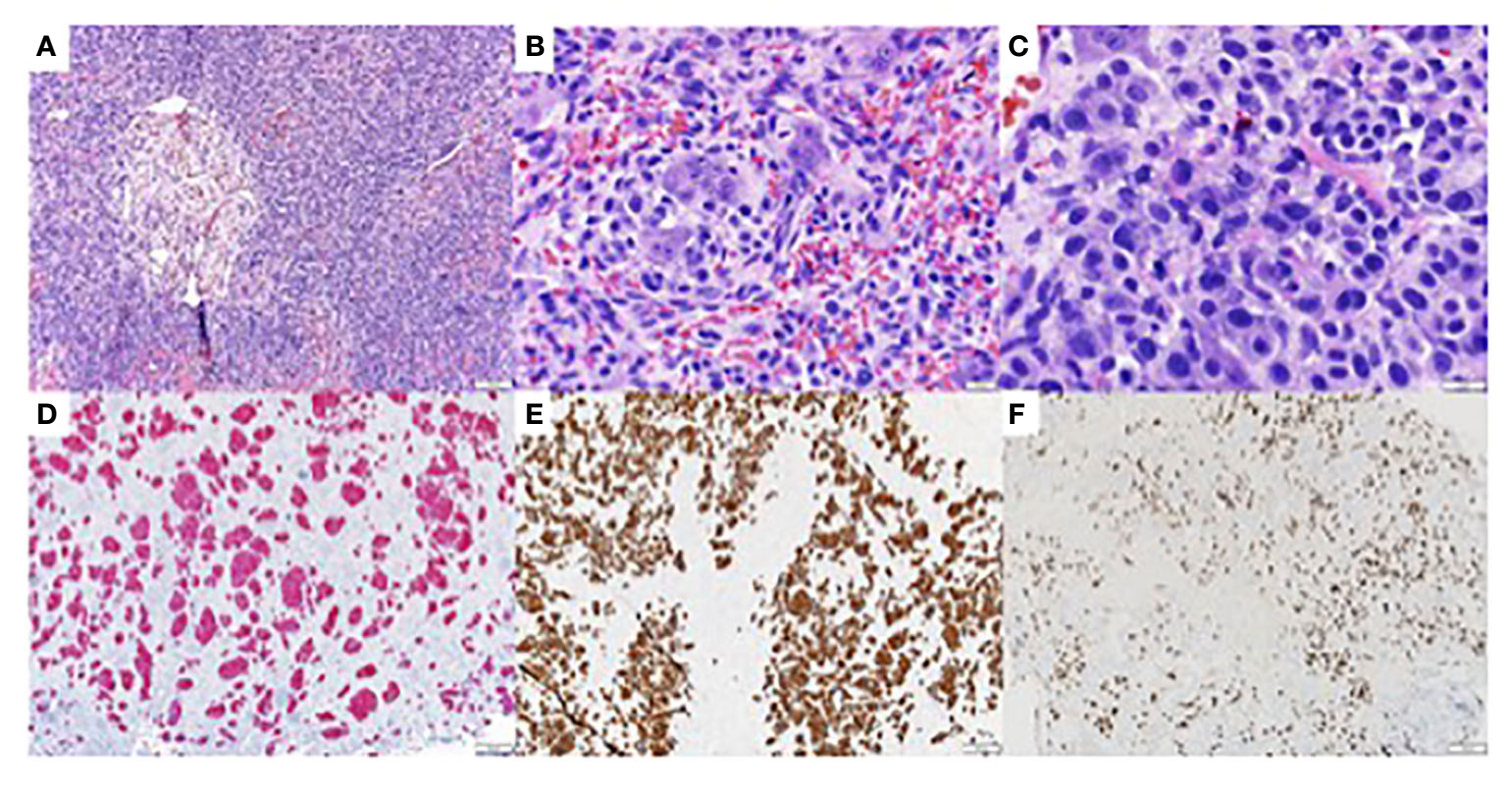
Immunohistochemistry studies of biopsy of metastatic tumor of unknown primary from the eighth and tenth thoracic vertebrae (T8 and T10) **(A)** Hematoxylin Eosin (H/E). 100x, showing clusters of large atypical cells with medium sized nucleoli and moderate amount of eosinophilic cytoplasm. **(B)** H/E, 400x **(C)** H/E, 600x **(D)** AE1/AE3 immunohistochemistry, 100x, showing diffuse and strongly positive staining of tumor cells. **(E)** CK7 immunohistochemistry. 100x, showing diffuse and strongly positive staining of tumor cells. **(F)** GATA-3 immunohistochemistry, 100x, showing diffuse and strongly positive staining of tumor cells.

Initially there was concern for multiple myeloma (MM), however serum protein electrophoresis and serum free light chains assays were negative for MM. Additionally, prostate-specific antigen was unremarkable. CT-guided biopsies of the T8 and T10 vertebral bodies showed findings consistent with metastatic, poorly differentiated carcinoma. Histopathology revealed that the neoplasm was comprised of clusters of large, atypical cells with medium-sized nucleoli and moderate amount of eosinophilic cytoplasm, with apocrine morphology. Immunohistochemistry (IHC) demonstrated that the cancer cells were strongly positive for pan-cytokeratin, cytokeratin AE1/AE3, CK7 and GATA-3 (**Figure 1**), and were negative for PD-L1 (TPS <1%), TTF-1, p40, S-100, p53, PSA, NKX3.1, PAX-8, desmin and CDX-2. A subset of cells showed coexpression of SATB-2 and p63. IHC was performed on FFPE sections on glass slides using automated staining techniques. Staining was scored for intensity (0 = no staining; ≥1 = weak; ≥2 = moderate; ≥2 = strong) and staining percentage (0% to 100%) by a board-certified pathologist (8). Overall, the immunophenotype was not able to confirm a site of origin, however, due to GATA-3 expression, primary sites under consideration included breast, salivary gland, and skin adnexa.

A molecular profiling assay (Caris Life Sciences, Phoenix, AZ) consisting of both whole-exome next-generation sequencing (NGS) as well as additional IHC for select clinically relevant proteins was performed on the bone biopsy. NGS (NextSeq or NovaSeq 6000, Illumina, Inc, San Diego, CA) was performed on genomic DNA isolated from microdissected, FFPE tumor samples using whole-exome sequencing (700 genes at high coverage and read depth). A hybrid pull down of baits designed to enrich for 700 clinically-relevant genes at high coverage and high read-depth was used. All genetic sequence variants were detected with >99% confidence based on allele frequency and amplicon coverage, with an analytic sensitivity of 5% and an average sequencing depth of coverage >500. Tumor enrichment was achieved by collecting targeted tissue using manual microdissection techniques prior to molecular testing. Genetic variants were interpreted by board-certified molecular geneticists and were classified as pathogenic, likely pathogenic, variant of unknown significance, likely benign, or benign according to the American College of Medical Genetics standards. For androgen receptor (AR) protein staining, a mouse anti-AR monoclonal antibody was used (AR441, Abcam, Cambridge, UK) and staining was considered positive if the intensity was ≥1+ on ≥10% of cells (8). Results of the assay suggested a triple-negative (ER/PR/ERBB2 negative, by IHC) and AR positive (IHC 2+) tumor profile. Other genetic biomarkers included mutations in *CDH1* (protein alteration p.R598*, DNA alteration c.1792C>T, variant allele frequency [VAF] 57%), *PTEN* (p.Q298*, VAF 23%), *TP53* (p.R209fs, VAF 20%), and *KMT2C* (p.Q1186*, VAF 15%).The tumor mutation burden was low (9 mut/Mb) and microsatellite status was stable.

The Genomic Prevalence Score (GPSai) revealed an 81% confidence for breast cancer; however, on clinical evaluation and advanced imaging, he had no signs of a breast neoplasm. In addition to the bony metastases, whole body FDG-labelled positron emission tomography-computerized tomography (PET-CT) revealed hypermetabolic lymphadenopathy and a large soft-tissue tumor in the upper left neck suggestive of the primary tumor. The radiographic and histopathologic features were consistent with carcinoma and excluded a hematopoietic process such as lymphoma. Given pathological fractures and aggressive disease biology (e.g., poorly differentiated carcinoma and metastatic disease), systemic treatment with carboplatin and paclitaxel were initiated to treat metastases of unknown primary carcinoma (9).

Germline testing later returned positive for a heterozygous *CDH1* pathogenic variant. Interestingly, the patient’s father had been diagnosed with gastric cancer in his 60s. Given germline *CDH1* pathogenic variant’s association with DGC, the patient underwent esophagogastroduodenoscopy which was negative for gastric malignancy. Initially he showed a partial response to treatment with carboplatin and paclitaxel, however he later developed disease progression after 6 months with increasing cervical and axillary lymphadenopathy.

Given disease progression with standard chemotherapy and IHC suggesting possible skin adnexa as primary site of disease, the patient was treated with off-label immunotherapy using pembrolizumab 200mg intravenously every 3 weeks. Prior studies in patients with advanced melanoma and cutaneous squamous cell carcinoma treated with pembrolizumab monotherapy had shown both improved overall survival and durable responses to therapy, respectively (10, 11). Thus, the goal was to achieve long-term disease control using off-label immunotherapy. However, his cancer progressed within 2 months with an increasing neck mass and new liver lesions.

**Figure 2:**
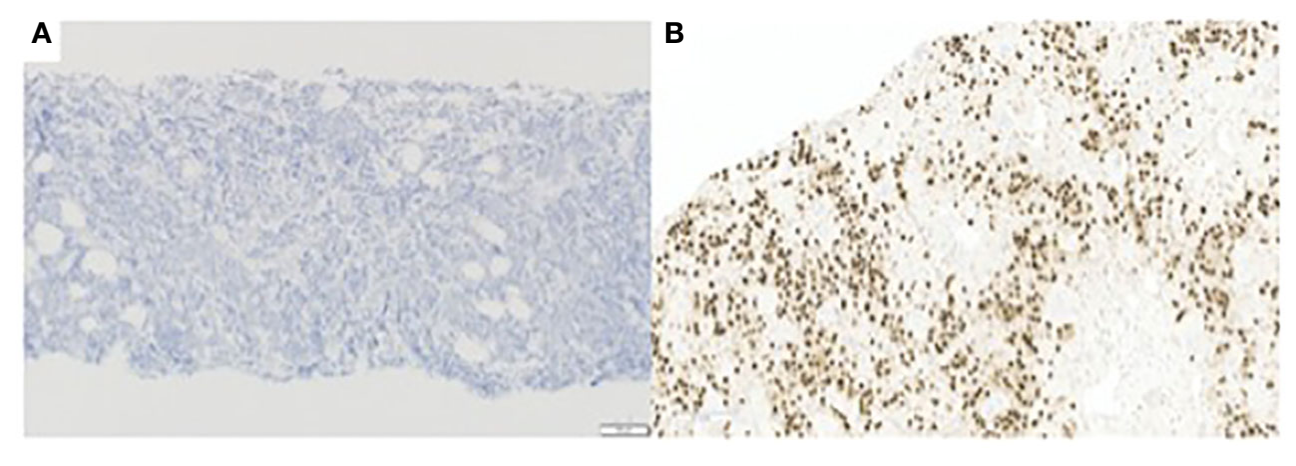
**(A)** E-cadherin immunohistochemistry, 100x, tumor cells are negative for E-cadherin. **(B)** AR immunohistochemistry. 200x, showing positive staining of tumor cells.

To newly evaluate histology and molecular alterations from a non-osseous source, a cervical lymph node from the left neck was biopsied and was again consistent with poorly differentiated carcinoma. IHC was positive for CK7, GATA3, and AR (**Figure 2**), which were similar to the previously diagnosed vertebral metastasis. Biopsy results suggested that the primary tumor origin was salivary gland in this patient’s clinical context given that salivary and mammary gland tumors share similar morphologic and genotypic profiles (12).

**Figure 3:**
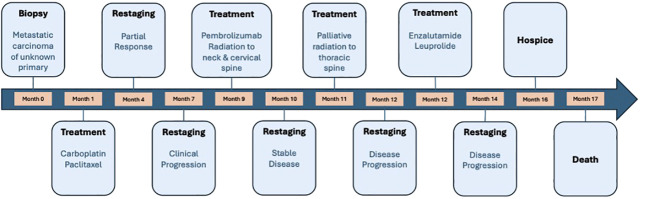
A timeline of the treatments our patient received throughout his treatment course. Time from the start of the first line of therapy to the occurrence of first relapse (PFS1) was 5 months and overall survival was 17 months.

He was then treated with leuprolide and enzalutamide given that the tumor was AR positive (by IHC) (13); however, his disease continued to progress despite AR-directed therapy. Due to the incurable nature of disease and rapid disease progression, the patient opted to transition to symptom-based management and enrolled in hospice care. Treatment was discontinued, and the patient died several weeks later. Overall survival was 17 months. Time from the start of the first line of therapy to the occurrence of first relapse (PFS1) was 5 months (**Figure 3**).

**Figure 4:**
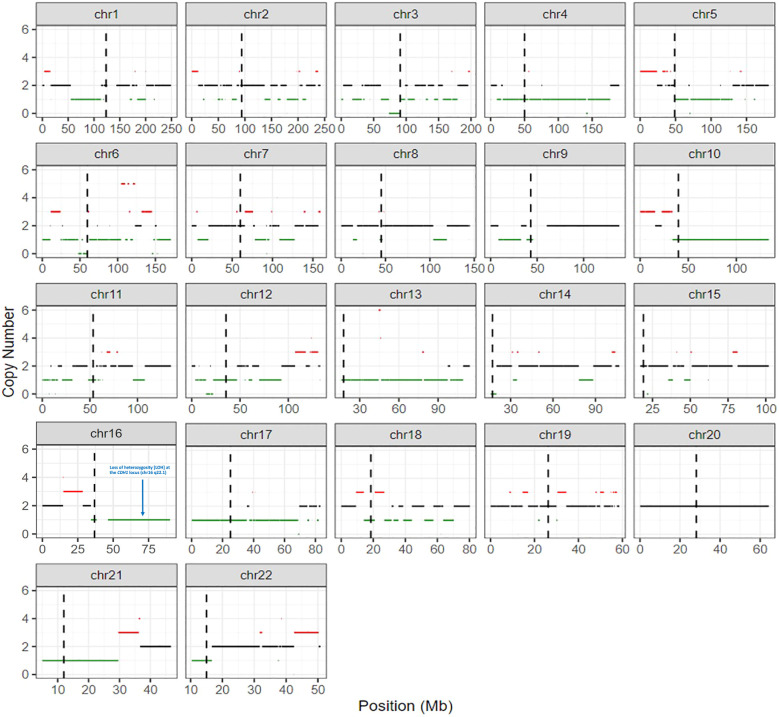
Molecular karyotype generated by detecting copy number gains and losses from next-generation sequencing data using CNVkit. More specifically, loss of heterozygosity (LOH) is seen on chromosome 16 at the *CDH1* locus (chr16 q22.1) indicating that our patient had bi-allelic inactivation of the *CDH1* gene.

In a post-mortem examination, a lymph node biopsy sample was analyzed, and a copy-number profile was generated to investigate whether there was loss of the *CDH1* allele. Copy number gains and losses were detected from NGS data using CNVkit (14). CNVkit normalizes read depths among on- and off- target sites for a given sample to a constructed reference. The method adjusts for GC content, target size, and sequencing repeats before producing absolute copy number calls in discrete segments consisting of multiple genes. The segmentation step (circular binary segmentation) involves an algorithm determining consistent depth data to make an accurate prediction. Copy number status per segment was generated, with copies for each allele and total copies, along with confidence metrics per segment. For the purposes of this analysis, the copy value and confidence metrics of a given gene are considered to be identical to the values of the segment that contains the gene. A gene was called indeterminate if the segment-level copies were ≤ 0.1 with the 95% prediction interval ≥ 1. These genome-wide copy number results were plotted graphically to generate the karyotype (**Figure 4**). This copy-number analysis showed LOH at the *CDH1* locus revealing that our patient had bi-allelic inactivation of the *CDH1* gene and demonstrating complete loss of E-cadherin protein expression (**Figure 4**). Furthermore, E-cadherin immunohistochemistry of the cervical lymph node biopsy showed that tumor cells were negative for E-cadherin (**Figure 2**).

## Discussion

We present a patient with a germline *CDH1* pathogenic variant and clinical features suggestive of metastatic SDC given histopathological apocrine features, AR positivity on molecular profiling, and imaging revealing a large soft tissue mass in the upper left neck suggestive of a primary salivary tumor. We propose that our patient is unique, as this is the first known case report of conventional SDC occurring in the setting of a germline *CDH1* pathogenic variant. As first proposed by Kusafuka et al., this case report supports the notion that SDC may be the salivary counterpart of LBC and DGC in individuals with germline *CDH1* pathogenic variants due to similarities in immunophenotyping studies and morphologic assessment (7).

When investigating the clinicopathologic features of patients with SDC-RF, Rooper et al. found that 8 of 9 patients were male and had a median age of 67 (range 63-83 years) (6). Similarly, Kusafuka et al. aimed to investigate somatic genetic changes in the *CDH1* gene in 17 patients with SDC-RF and found that 13 of the patients were male and had a mean age of 62. Primary sites of cancer included the parotid gland, accessory parotid gland, submandibular gland, and pharynx; there was one case of metastatic carcinoma of unknown origin. Due to significant cervical lymphadenopathy, fifteen patients underwent neck dissection and 13 of the patients were found to have metastases. Tumor resection or lobectomy was completed in fourteen patients, while additional postoperative radiotherapy or chemoradiotherapy was completed in 6 of these patients. By the end of the study, seven patients had died from SDC-RF, while 8 patients were living with metastases to bone, lung, and liver (7). Clinical characteristics of patients with SDC described by Kusafuka et al. and Rooper et al. are similar to our patient in terms of average age of diagnosis, presence of significant cervical lymphadenopathy, presence of metastases to the lung and liver, and the aggressive nature of disease as noted by rapid progression despite systemic treatment with chemotherapy, immunotherapy, and AR-directed therapy.

Recently, the literature has aimed to refine criteria for diagnosing SDC. Both AR positivity and apocrine morphology resembling comedo type of either breast ductal or lobular carcinoma in-situ are now considered central to establishing the diagnosis. Histopathologic examination by Rooper et al. revealed that all tumor cells of patients with SDC-RF were overtly rhabdoid, AR positive, and had an apocrine appearance with prominent nucleoli and abundant eosinophilic cytoplasm (6). In contrast, histopathologic examination of our patient’s tumor showed moderate eosinophilic cytoplasm, medium-sized nucleoli, AR positivity without overtly rhabdoid shaped cells, suggesting a diagnosis of conventional SDC as opposed to SDC-RF.

While not essential to diagnose SDC, the presence of *CDH1* alterations in SDC-RF has been observed. Molecular analysis by Rooper et al. and Kusafuka et al. found that greater than 70% of cases of SDC-RF had somatic *CDH1* alterations. Six of the 7 cases that underwent targeted NGS by Rooper et al. showed *CDH1* alterations of which monoallelic mutations were present in 4 cases and biallelic inactivation was present in 2 cases. Alteration types included single copy deletions, frameshift mutations, nonsense mutations, and splice-site mutations (6).

Furthermore, Kusafuka et al. was able to conduct somatic genetic analysis of the *CDH1* gene in 12 cases of which 6 cases exhibited missense/nonsense mutations in exon 2. Other alterations seen included an insertion in exon 2, and missense mutations in exon 3,4,7,14, and 16. Somatic mutations in the *CDH1* gene were seen in more than half of the cases of SDC-RF and this correlated with loss or aberrant expression of E-cadherin protein in 83% of cases (7).

Unlike the prior studies mentioned above, germline testing in our patient revealed a nonsense mutation (p.R598*, DNA alteration c.1792C>T) in exon 12 of the *CDH1* gene, which is unique in patients with SDC. Traditionally, this variant is believed to result in a premature stop codon leading to absent or incomplete protein and has been reported to occur in families with hereditary DGC (15). Further tumoral molecular testing revealed the VAF of the *CDH1* alteration was 57%. The tumor content was 65% in the H&E. Since the patient has a known germline *CDH1* pathogenic variant, a VAF of ~60% would imply allele-specific loss of heterozygosity (LOH) at the 16q22.1 locus. The karyotype (**Figure 4**) shows LOH at the *CDH1* locus, confirming that our patient had bi-allelic inactivation of the *CDH1* gene.

In addition to LOH at the *CDH1* locus, we observed multiple additional areas of shallow (hemizygous) chromosomal deletions throughout the genome, plus three areas of deep (homozygous) deletions involving chromosome 3p (peri-centromeric), chromosome 6p (peri-centromeric) and chromosome 12p. These hemizygous and homozygous genomic losses are of unclear clinical significance. The overall degree of genome-wide loss of heterozygosity (gLOH) was 8%, consistent with a modest level of copy number alterations and below the 16% threshold that defines high gLOH.

IHC of a cervical lymph node biopsy also showed that the tumor cells were negative for E-cadherin protein (**Figure 2**). We believe that the NGS results are consistent with the IHC results in our patient. With respect to *CDH1*, we observed a truncating mutation as well as genomic loss of the wild-type allele, consistent with *CDH1* (E-cadherin) protein loss seen in the immunohistochemical studies. Additionally, with respect to *TP53*, it is thought that only the dominant-negative (gain-of-function) *TP53* missense mutations result in nuclear p53 protein accumulation, not those predicted to be truncating (16). The observed *TP53* mutation in our patient is a frameshift mutation that results in a truncated transcript and protein. Thus, it is not associated with nuclear accumulation or a positive p53 IHC result.

We believe that our patient developed a somatic *CDH1* loss in addition to his preexisting germline *CDH1* mutation that ultimately resulted in loss of E-cadherin protein predisposing him to SDC. While previous studies have found somatic *CDH1* pathogenic variants in SDC-RF, our patient was found to have a germline *CDH1* pathogenic variant in the setting of conventional SDC, without rhabdoid features. It is important to note that there may be other genetic alterations, molecular factors, and pathways contributing to the development of SDC that were not investigated in this study. For example, the NGS assay used is not capable of interrogating epigenetic factors such as gene methylation or acetylation, and it is possible that epigenetic silencing of unknown genes could be important. The assay also failed to interrogate non-coding regions of the genome (introns, intergenic regions, gene enhancers, promoters) as well as the telomeres and centromeres. In addition, the panel of IHC markers was limited, and we did not undertake an exhaustive analysis of other potential protein losses. Future research could aim to understand additional factors that may predispose patients with germline *CDH1* pathogenic variants to develop SDC.

In conclusion, we report a unique case of a patient with SDC found to harbor a germline *CDH1* pathogenic variant with somatic biallelic inactivation. This case provokes questions regarding tumor genetics and molecular profiling of SDC in patients with germline *CDH1* pathogenic variants. Moreover, this case supports the notion that SDC may be the salivary counterpart of LBC and DGC in individuals with germline *CDH1* pathogenic variants and may possibly expand the spectrum of tumors that arise in this familial cancer-predisposition syndrome.

## Data availability statement

The original contributions presented in the study are included in the article/supplementary material. Further inquiries can be directed to the corresponding author.

## Ethics statement

Written informed consent was obtained from the patient for the publication of any potentially identifiable images or data included in this article.

## Author contributions

ND: Investigation, Writing – original draft, Writing – review & editing. ER: Data curation, Writing – review & editing. NF: Writing – review & editing. AG: Supervision, Writing – original draft, Writing – review & editing. ESA: Methodology, Supervision, Writing – review & editing.
